# Traumatic segmental renal infarction due to renal apical artery thrombosis by minor blunt abdominal trauma: the role of CEUS

**DOI:** 10.1007/s40477-023-00817-0

**Published:** 2023-08-23

**Authors:** Rosita Comune, Francesca Grassi, Stefania Tamburrini, Carlo Liguori, Fabio Tamburro, Salvatore Masala, Mariano Scaglione

**Affiliations:** 1 Division of Radiology, Department of Precision Medicine, University of Campania Luigi Vanvitelli, Piazza Miraglia 2, 80127 Naples, Italy; 2 Department of Radiology, Ospedale del Mare-ASL NA1 Centro, Naples, Italy; 3 Department of Medicine, Surgery and Pharmacy, University of Sassari, Piazza Università, 21, 07100 Sassari, Italy; 4 Department of Radiology, James Cook University Hospital, Marton Road, Middlesbrough, TS4 3BW UK

**Keywords:** Renal trauma, Apical renal artery thrombosis, Vascular injury, Blunt abdominal trauma

## Abstract

Post-traumatic segmental renal infarction is an extremely rare event, especially in case of minor blunt abdominal trauma. While major trauma guidelines are well established, several problems account for the adequate management of minor trauma. Herein, we report a case of minor blunt abdominal trauma determining traumatic thrombosis of the apical renal artery and segmental renal infarction, firstly diagnosed by CEUS in emergency care setting.

## Introduction

Renal injury accounts for approximately 1–5% of all trauma admissions and as many as 10% of patients who sustain abdominal trauma [1–3]. However, injury to renal vessels represents a rare complication of blunt abdominal trauma, which was firstly described by Von Recklinghausen in 1861. Particularly, artery or vein laceration and avulsion are the most commonly vascular injuries described, while thrombotic renal artery occlusion, after a blunt abdominal trauma, is an extremely rare event, and it represents less than 1% of renal vascular injuries [4–7]. Due to the rarity of these thrombotic injuries, to date both diagnostic and therapeutic algorithms are still lacking. Herein, we report a case of minor blunt abdominal trauma determining traumatic thrombosis of the apical renal artery and segmental renal infarction, firstly diagnosed by CEUS in emergency care setting.

## Case presentation

A 21-year-old male patient referred to our Emergency department with a history of minor blunt abdominal trauma sustained after falling off a bunk bed. The patient complained of pain localized to the left lumbar and hypochondriac region. No external wound or ecchymosis was noted at physical examination.

The patient was hemodynamically stable (pulse rate 100/min, blood pressure 110/60 mmHg). Laboratory tests were unremarkable (hemoglobin: 14 g/dl, normal value 11.5–17.5 g/dl, PCR: 5 mg/dl normal value 0.0–0.5 mg/dl), no macro or microhematuria was detected.

**Fig. 1:**
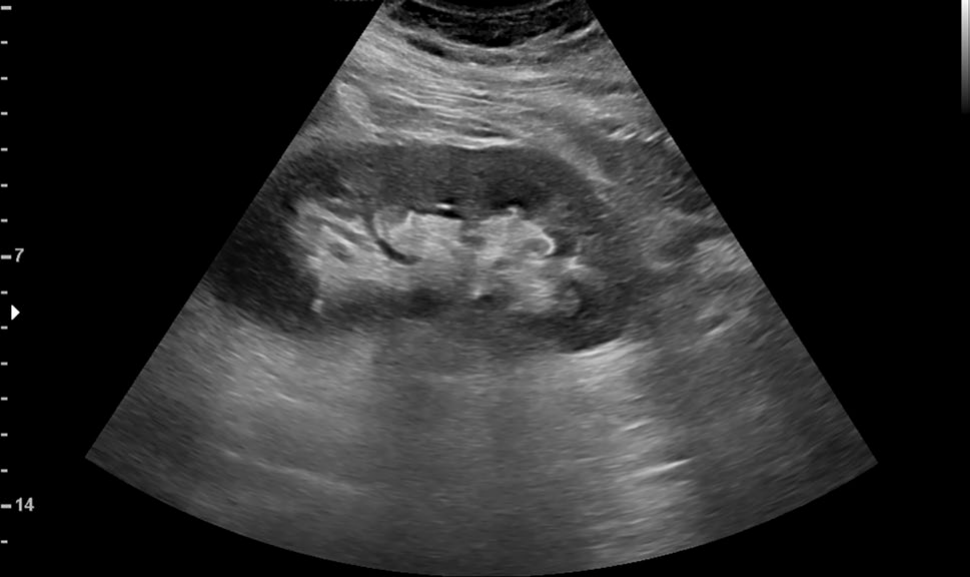
B-mode ultrasound. Left kidney with a normal cortical medullary representation of the parenchyma. No perirenal fluid or subcapsular hematoma were appreciable. Non parenchymal lesions or intraparenchymal fluid collections were detected

Because of the absence of any criteria for major abdominal trauma (dynamic, anatomical, physiological) and no risk factors [8, 9], the patient underwent ribs X rays and E-FAST ultrasound examination. The X-rays showed no ribs fractures. The E-FAST examination was performed by a radiologist and was negative (no pleural or pericardial effusion, no free fluid in the abdomen, and preserved sliding sign in the lung). Because of the intense pain in the left hypochondriac region and in order to improve the sensitivity of the FAST examination, supplementary ultrasound views were performed (subdiaphragmatic, caudal liver margin, paracolic groove, between intestinal loops, retroperitoneal and right upper abdomen), as well as a complete abdominal B-mode examination, in order to identify any solid organ lesions without hemoperitoneum [10] (Fig. 1). During the examination, the patient complained of a sudden stabbing pain at the left hypochondriac region, referring even a worsening of his symptoms. Hence, a CEUS examination was performed to evaluate the presence of any renal or splenic parenchymal lesions not appreciable at B-mode. Contrast enhanced ultrasound was performed (2.4 ml of Sonovue followed by 10 ml of saline injected manually through an antecubital vein on the right arm).

**Fig. 2:**
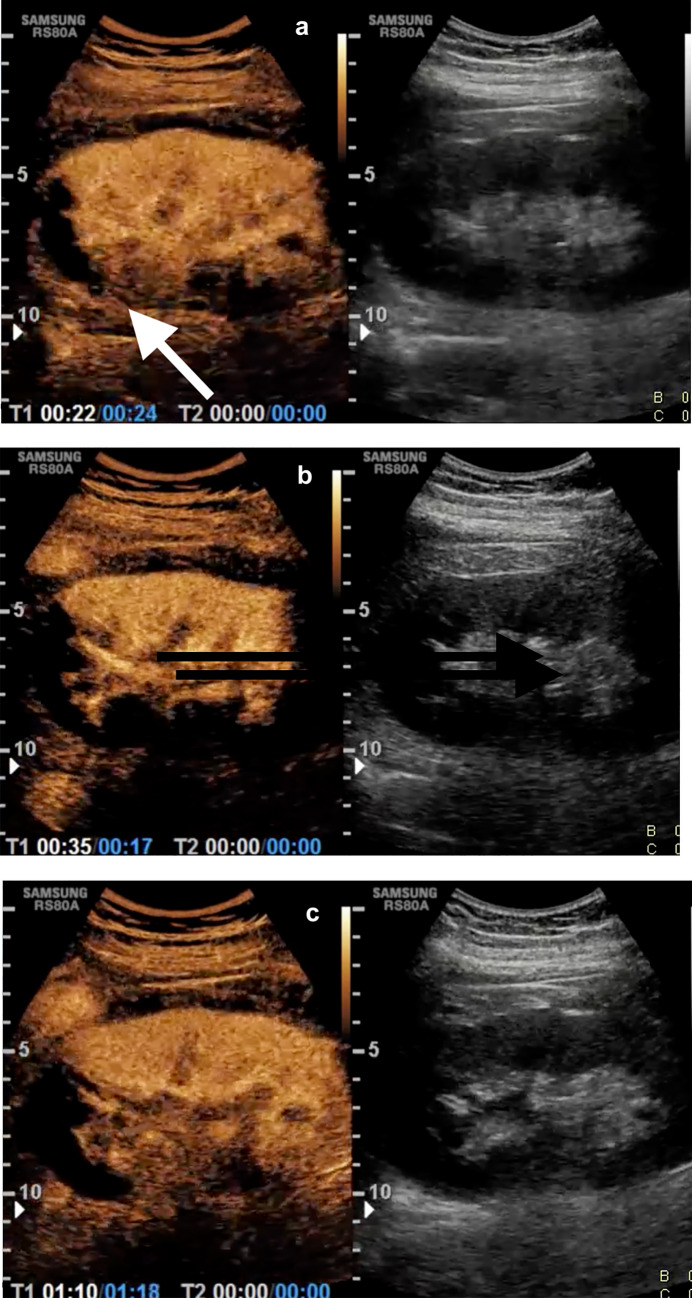
CEUS examination. **A** At 22 s after the administration of Sonovue, intravascular distribution of microbubbles was detected in the entire renal cortex with the exception of a band-like area in the upper pole of the left kidney (white arrow). **B** At 35 s after contrast agent administration, the band-like area in the upper pole of the left kidney was persistently avascular. **C** At 70 s. The persistent and homogeneous appearance of the avascular area with absence or rim sign was suspicious of non-complicated segmental renal infarction

**Fig. 3:**
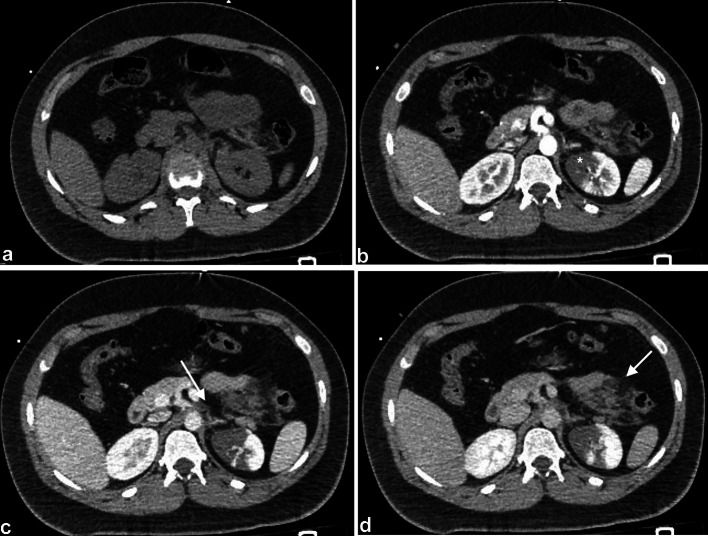
CT axial without contrast (**A**), during the arterial phase (**B**) nephrographic phase (**C**), excretory phase (**D**). In the upper pole of the left kidney appeared, a well-demarcated area of reduced/absent contrast enhancement in all phases was detected (**B** white asterisk). Non perirenal fluid or subcapsular hematoma were present there was a mildly fat stranding in the para-aortic region (**C** ached arrow). Moreover, CT showed mesenteric fat standing in left hypochondriac region suggestive of mesenteric hematoma without active bleeding or bowel perforation (white arrow)

**Fig. 4:**
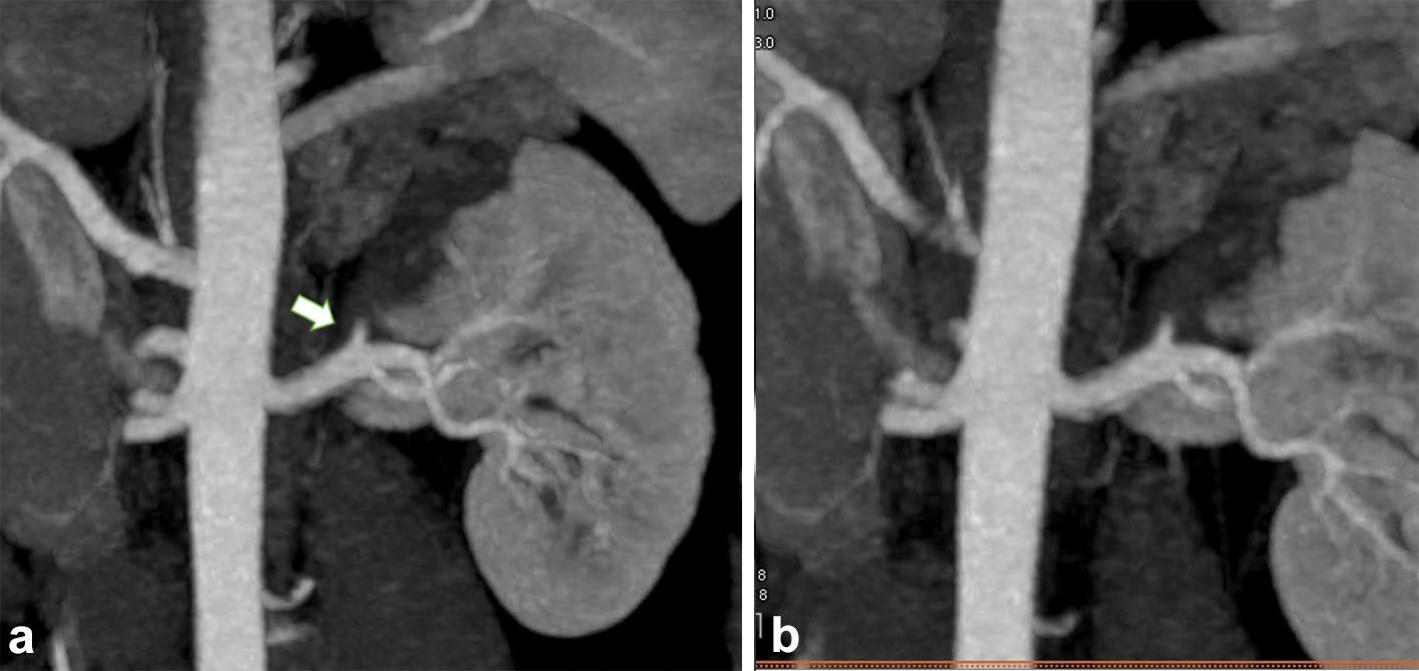
CT coronal **A**, **B** views shows a faintly perfused left kidney and thrombotic occlusion of the segmental apical renal artery (white arrow)

After microbubbles were administered intravenously, a cortico medullary anechoic band-like area was immediately detected at the upper pole of the left kidney and after 32 s the absence of enhancement was persistent and homogenous. At 45 s, the upper pole remained avascular (Figs. 2, 3).Based on positive CEUS findings, a suspected diagnosis of segmental left kidney avascular lesion was formulated at CEUS. The patient, based on positive CEUS findings underwent abdominopelvic CT with intravenous contrast (1.0–1.5 mL/kg injected at 3.5 mL/s, followed by 50 mL of a saline bolus injection). A CT multiphase protocol was acquired (non contrast, arterial, corticomedullary, parenchymal and excretory phases) (Fig. 3). CT showed left renal vascular injury with segmental infarct appearing as a coarse, sharply defined, band-like area at the upper pole of the left kidney (Fig. 3). Non perirenal fluid or subcapsular hematoma were present there was a mildly fat stranding in the para-aortic region. The hypoperfusion was secondary to thrombotic occlusion of the left renal apical artery (Fig. 4a, b). The patient was hemodynamically stable with no evidence of micro or macrohematuria. For the next 72 h, so revascularization was not attempted, and close observation and conservative treatment was achieved. Every 4 h, blood pressure was monitored, and patient underwent medical therapy (enoxaparin 4000 UI). Because of the presence of a vascular renal lesion a follow-up CT was performed after 24 h, that showed stability of findings. A follow-up CEUS was performed and confirmed the segmental renal infarct with no signs of complications. The patient was discharged after 2 weeks.

## Discussion

E-FAST is a useful bedside tool for ruling in pneumothorax, pericardial effusion, and intra-abdominal free fluid in the trauma setting. However, FAST examination is an imperfect test. Indeed, its sensitivity is extremely variable between 41 and 95%, and has not to be used to rule out intra-abdominal injuries [11–15]. Thus, in hemodynamically stable patients with major trauma, a CT scan is recommended whether the FAST scan is positive or negative, and according to Trauma Guidelines, E-FAST should be reserved for hemodynamically unstable patients [10–13, 16, 17]. Meanwhile, major trauma guidelines are well established, several problems account for the adequate management of minor trauma.

Patients with minor trauma are usually managed with FAST examination and segmental x rays, depending on clinical symptoms. In case of negative e-FAST, and with no clinical or laboratory alterations during the observation period, patients are usually dismissed. However, even in cases of negative FAST examinations, in patients where a clinical or laboratory alterations may lead to the persistence of any suspicion of misdiagnosed conditions, repeated laboratory analysis, and serial FAST examination are required. At serial ultrasound examination, additional ultrasound view and B-mode examination of parenchyma are performed to increase ultrasound sensitivity [10, 16]. However, especially for minor abdominal trauma, diagnostic approved flow-chart is still missing, and the choice of additional diagnostic examinations is based on the clinical presentation and physician experience.

We reported an interesting case of a multimodality approach to minor blunt abdominal trauma in a young patient. Particularly, our case underlines the CEUS's role in minor trauma. CEUS can be extremely useful in screening patients requiring second-level imaging, reporting no alterations at the E-FAST examinations. It may represent a problem-solving imaging method and a speeding-up modality for the prompt management of patients.

Particularly, although more studies are needed to confirm these results, CEUS examination may be proposed as first-line imaging in patients with a negative FAST examination reporting focused symptoms after minor blunt abdominal trauma. Indeed, the diagnostic value of CEUS has been showed to be higher than the conventional ultrasound examination [18]. Notably, CEUS can reveal parenchymal lesions without hemoperitoneum or subcapsular hematoma [18–20], leading to an accurate definition of the parenchymal damage, as in our case, demonstrating the avascular renal parenchyma [20] suspicious for renal trauma.

Renal trauma is responsible for 30.8% of acute renal infarction, meanwhile, post-traumatic segmental renal infarction is an extremely rare event. Segmental renal infarction has been reported secondary to cardiogenic and systemic conditions or iatrogenic causes, traumatic segmental renal infarction secondary to a thrombosed segmental artery is an exceptional event [21].

The rate of thrombosis of the renal artery after renal trauma is estimated around 2% [22], and isolated segmental kidney infarction is classified as grade IV injuries, although the classification does not take into account the heterogeneity of this lesion and the bleeding risk, in fact, it has been proposed to reclassify isolated kidney infarction as grade III injury [22].

Avascular lesions, such as infarcts, are confidently identified at CEUS examination; infarcts appear on CEUS as wedge-shaped areas showing no enhancement. In our case, the avascular lesion presented a band-like morphology. The band-like appearance of the infarct was due to a segmental apical artery according to pre-segmental anatomical branching of renal arteries, the knowledge of segmental and pre-segmental renal artery branching is important to correctly identify vascular lesions, which may present different wedge-shaped or band-like morphologies, depending on the arterial distribution.

In our case, we were not able to identify the cortical rim signs, probably due to the cute and traumatic event. However, MDCT with intravenous contrast confirmed the infarcted area on the upper pole of the left kidney, identifying the renal pre-segmental artery branching and the traumatic thrombosis of the apical artery.

## Conclusion

Our case demonstrated the potential central role that CEUS examination may have in the management of minor blunt trauma in clinically symptomatic patients with a negative FAST examination. However, more studies are needed to confirm the role of CEUS in these emergency situations. Post-traumatic Segmental Renal Infarction due to Renal Apical Artery Thrombosis is an exceptional event and the knowledge of renal artery segmentation is important to characterize the avascular region. In our case, CEUS correctly identified the renal infarct, and justified the second-level imaging with MDCT with intravenous contrast that accurately demonstrated the renal artery branching and apical artery thrombosis. Hence, CEUS examination may represent a first-line diagnostic tool to improve the correct selection of patients needing second line examinations, improving the diagnostic sensibility of clinically symptomatic patients showing no alterations at FAST examination. However, more studies are needed to confirm our results, and to clarify the potential role of CEUS.

## Data Availability

The data that support the findings of this study are available from the corresponding author, [RC], upon reasonable request.
